# How Does Physical Activity Affect the Mental Health of Adults with Intellectual Disability? A Cross-Sectional Study Analyzing the Complex Interplay Between Variables

**DOI:** 10.3390/jfmk10030285

**Published:** 2025-07-24

**Authors:** Tommaso Piva, Andrea Raisi, Valentina Zerbini, Sabrina Masotti, Erica Menegatti, Alessandro Grande, Giovanni Grazzi, Gianni Mazzoni, Emilio Paolo Visintin, Martino Belvederi Murri, Simona Mandini

**Affiliations:** 1 Center for Exercise Science and Sport, Department of Neuroscience and Rehabilitation, University of Ferrara, 44121 Ferrara, Italy; tommaso.piva@unife.it (T.P.); andrea.raisi@unife.it (A.R.); sabrina.masotti@unife.it (S.M.); erica.menegatti@unife.it (E.M.); alessandro.grande@unife.it (A.G.); giovanni.grazzi@unife.it (G.G.); simona.mandini@unife.it (S.M.); 2 Department of Environmental Sciences and Prevention, University of Ferrara, 44121 Ferrara, Italy; 3 Healthy Living for Pandemic Event Protection (HL-PIVOT) Network, Chicago, IL 60607, USA; 4 Public Health Department, AUSL Ferrara, 44121 Ferrara, Italy; 5 Department of Humanities, University of Ferrara, 44121 Ferrara, Italy; emiliopaolo.visintin@unife.it; 6 Institute of Psychiatry, Department of Neuroscience and Rehabilitation University of Ferrara, 44121 Ferrara, Italy; martino.belvederimurri@unife.it

**Keywords:** intellectual disabilities, anxiety, depression, physical activity, sport

## Abstract

**Background:** Individuals with intellectual disability face an increased risk of mental health issues compared to the general population. Despite the proven efficacy of physical activity (PA) in improving anxiety and depression in the general population, little is known about this relationship in adults with intellectual disability and the factors that influence it. The purpose of the study was to determine whether a correlation exists between PA and perceived levels of anxiety and depression, and assess gender disparities in PA and mental health. **Method:** People with intellectual disability were recruited from day centers and sports events. The amount of PA was evaluated through the International Physical Activity Questionnaire. Perceived mental health was assessed through the Zung Self-Rating Anxiety (ZAS) and Depression Scale (ZDS). **Results:** In total, 99 adults (34 females, aged 33 ± 12) participated in the study. A gender disparity was found in anxiety levels, while depression, PA, and type of sport participation did not differ between males and females. Multiple regression analysis highlights how the depression score was significantly predicted by gender (β = −3.57, *p* = 0.015), intellectual disability level (β =−3.08, *p* < 0.008), and PA (β =−0.10, *p*= 0.001), while anxiety was influenced by gender (β = −4.48, *p* = 0.003) and intellectual disability level (β = −3.23, *p* = 0.007). **Conclusions:** These findings underscore the relevance of physical activity as a factor associated with lower depressive symptoms in adults with intellectual disability, highlighting its potential role in mental health promotion within this population.

## 1. Introduction

Intellectual disability, defined as “a significantly reduced ability to understand new or complex information and to learn and apply new skills”, constitutes a group of neurodevelopmental disorders characterized by intellectual functioning and adaptive behavior limitations [1]. According to the literature, intellectual disability begins before adulthood, resulting in a reduced ability to cope independently, with a lasting effect on development [1]. It can cause severe limitations in intellectual functioning and adaptive behavior, and has the potential to impact an individual’s cognitive, social, and emotional dimensions [2]. The prevalence of intellectual disability across the world is estimated to be around 1%, with a male-to-female ratio varying between 0.7 and 0.9 [3]. Compared to the typically developing population, individuals with intellectual disabilities experience many more health issues [4,5,6]. Particularly, they are more likely to experience psychological disorders, with an estimated 12-month prevalence of 33.6% [7], compared to 17.6% in the general population [8], representing a serious public health concern. Research has increasingly highlighted the necessity of addressing mental health difficulties in people with intellectual disability, as enhancing their general well-being is critical for supporting a satisfying and meaningful life [9]. Comprehending the emotional state and the mental health perception of individuals with intellectual disabilities appears challenging, given the subjective nature of their understanding, awareness, and assessment of these conditions. Previous studies delved into predictors of mental health status in adults with intellectual disability, identifying the role of socio-demographic variables like support, accommodation, parental divorce, and adult abuse as significant predictors [10]. Particularly, anxiety and depression are among the most common psychiatric conditions in both the general population and individuals with intellectual disabilities. These disorders are associated with substantial functional impairment and are often underdiagnosed or undertreated in people with intellectual disabilities due to communication difficulties and limited access to appropriate mental health services [11].

Engagement in physical activity (PA) and sports interventions has a positive impact on the psychological well-being of individuals with intellectual disability, specifically mitigating stress, anxiety [12], and depression [13], and enhancing strength, cardiovascular fitness, and muscular endurance [14,15,16]. However, the prospective role of PA in determining mental health status in adults with intellectual disability remains unexplored in cross-sectional and epidemiological studies, despite the literature highlighting the existence of gender disparities in both mental health and PA levels [17,18].

Recent systematic reviews [19,20,21] have demonstrated that physical activity interventions significantly enhance psychological well-being, quality of life, and mental health outcomes in adults with intellectual disability, supporting the rationale for investigating PA–mental health associations in this population.

There is a gap in knowledge regarding how these factors may interact within this population. In particular, beyond investigating the relationship between physical activity (PA) and affect (AF), there is a lack of studies examining how this relationship varies according to gender. In light of these considerations, the central hypothesis of this study is that there is a relationship between PA and mental health in adults with intellectual disabilities.

The first objective of the study was to determine whether a correlation exists between PA and perceived levels of anxiety and depression in adults with intellectual disabilities.

The second objective was to explore whether gender interacts with PA in shaping the association with mental health outcomes, specifically anxiety and depression. The third objective was to identify which individual characteristics, including gender, age, level of intellectual disability, and type of sport participation, are associated with levels of anxiety and depression in this population.

## 2. Materials and Methods

This is a cross-sectional study conducted on adults (aged 18 or older) with intellectual disability diagnosed according to DSM-V [1]. Participants were recruited via word of mouth in day centers for people with intellectual and relational disabilities and during national and international sporting events carried out in Italy. To reduce potential self-selection bias, the study was advertised in a neutral manner, without disclosing the researchers’ expectations or hypotheses regarding the relationship between physical activity and mental health. Data were collected between October 2021 and November 2022 by a trained healthcare professional and a kinesiologist. Each participant completed a questionnaire aimed at assessing PA, followed by an anxiety and depression scale. Anthropometric and anamnestic data like height, weight, type, and level of intellectual disability were self-reported, and participants were instructed to provide this information based on the most recent medical documentation. To be recruited for the study, each participant must not have been diagnosed with a mental disorder according to DSM-V and should not be under any antipsychotic pharmacological therapy. People with physical impairments or absolute medical contraindications that prevent participation in any sort of physical exercise were not considered eligible for the study. The study was approved by the Ethics Committee of the University of Ferrara (CSSM-2021), and written informed consent was obtained from all the participants.

### 2.1. Perceived Anxiety and Depression

The perceived mental health of the participants was assessed through the Italian version [22] of the Zung Self-Rating Anxiety and Depression Scale [23,24], adapted for people with intellectual disability [25,26]. Both are standard assessment instruments with good internal consistency and validity, and they encompass most DSM-V criteria for anxiety and depression [27,28]. Both questionnaires contain 20 items, which are scored using a 4-point Likert scale, and total scores range from 20–80.

The Zung Depression Score (ZDS) and Zung Anxiety Score (ZAS) were calculated as the sum of the scores of each item. The ZAS and ZDS showed an acceptable level of internal reliability (ZAS: Cronbach’s ⲁ = 0.76, 95% CI 0.70–0.83; ZDS: Cronbach’s ⲁ = 0.82, 95% CI 0.77–0.87).

### 2.2. Physical Activity Levels

PA levels were measured through the Italian version of the International Physical Activity Questionnaire (IPAQ) in short format [29]. The questionnaire consists of six questions to record the number of days (frequency) and the number of minutes per day (min/day) of participation in all kinds of vigorous, moderate, and walking physical activities during the last seven days. According to the recommendations of the IPAQ committee [30], a PA score was calculated and expressed in MET-min per week (min/week). The weekly amount of PA performed was obtained by multiplying the number of weekly minutes by a coefficient of 3 for moderate-intensity PA and by a coefficient of 7 for vigorous-intensity PA. Type of sport engagement was collected at the end of the IPAQ with an open-ended question and subsequently categorized as “Participant in Team Activity Sports or (PTS)” or “Participant in Individual Activity or Sports (PIS)” based on the type of sport activity carried out. IPAQ has been found to be a valid and reliable measure of PA in people with intellectual disability [31].

### 2.3. Statistical Analysis

For descriptive statistics, data are presented as mean ± standard deviation. An a priori sample size of 92 participants was calculated to fit a regression analysis able to detect a f^2^ = 0.15, categorized as medium, with a statistical power of 0.8 and an α value of 0.05, considering 5 predictors. Therefore, prior to analysis, normal distribution was explored for each variable using the D’Agostino–Pearson test. Differences between groups were assessed for continuous variables with normal distribution with Student’s *t*-test for independent samples, when the assumption of normality was not met, non-parametric alternatives were applied: the Wilcoxon rank-sum test for continuous variables, Fisher’s exact test for binary variables, and Pearson’s chi-squared test for categorical variables. All test results for normality are reported in the Supplementary Materials. Two multiple linear regression models were used to verify the association between PA and perceived anxiety and depression, and the strength of the association was verified through Spearman’s rank correlation coefficient. To formally assess whether the association between physical activity (PA) and mental health outcomes differed by gender, two ANCOVA models were fitted with PA as the covariate, gender as the fixed factor, and the PA × sex interaction term. Model 1 used the ZAS as the dependent variable; Model 2 used the ZDS. A theoretical causality model based on a directed acyclic graph (DAG) was developed according to the exposure variable (PA), outcome (anxiety and depression), and confounding variables (age, gender, and intellectual disability level), using the online software Dagitty, version 3.2.

We assumed that there would be a direct pathway (association) between PA and anxiety and depression. Furthermore, we assumed that age, gender, and level of intellectual disability could directly influence PA, anxiety, and depression. These assumptions aligned with the aforementioned literature, which showed that female gender, age, and intellectual disability level were associated with a higher risk of developing mental health issues [10].

To avoid unnecessary adjustment, spurious associations, and estimation errors, the backdoor criterion was used to select a minimum set of confounding variables to fit the analyses [32]. Two multiple linear regressions were performed for the variables indicated by DAG. The level of statistical significance was adjusted for multiple tests according to Benjamini–Hockberg [33]. Statistical analyses were performed using R Statistical Software 4.5.1 [R Core Team. A language and environment for statistical computing. Published online 2021. https://www.R-project.org/] [.

## 3. Results

### 3.1. Participant Characteristics

The study included 99 participants with available data for analysis. Participant characteristics divided by gender are summarized in Table 1.

### 3.2. Relationship Between PA and Mental Health

**Figure 1:**
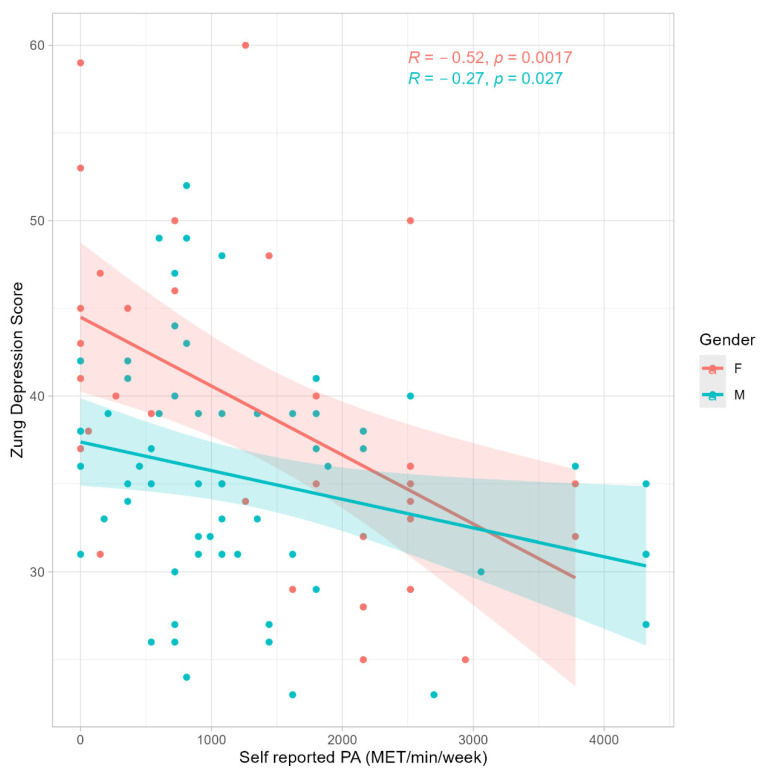
Pearson’s correlation between Zung Depression Score and self-reported physical activity expressed as metabolic equivalent (3.5 mL/kg/min of VO_2_) per minute of activity per week.

**Figure 2:**
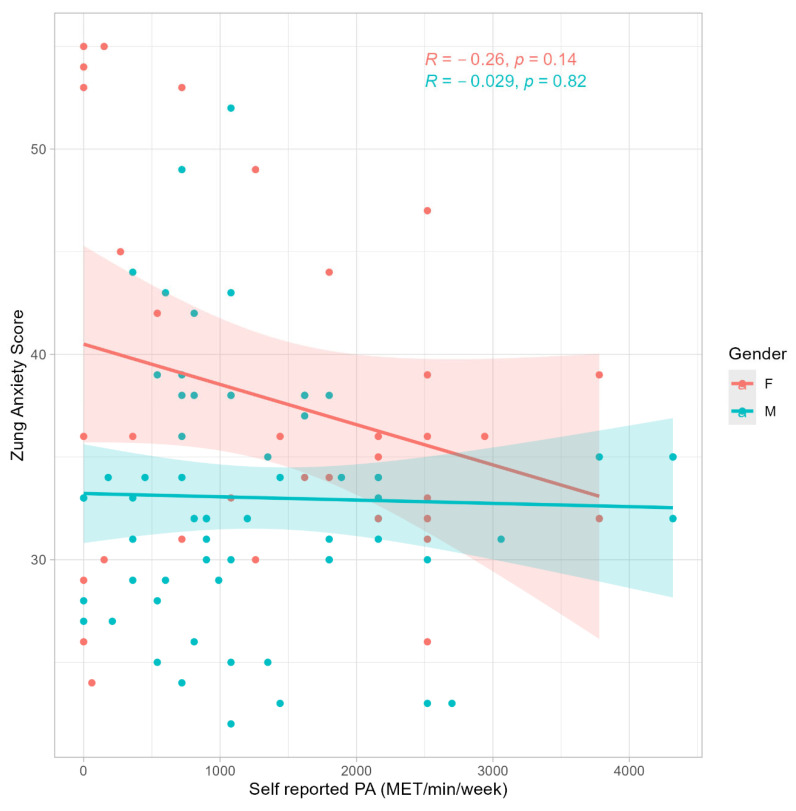
Pearson’s correlation between Zung Anxiety Score and self-reported physical activity expressed as metabolic equivalent (3.5 mL/kg/min of VO_2_) per minute of activity per week.

A statistically significant correlation was detected between the ZDS and PA across the entire sample (R = −0.35, *p* < 0.001) (Figure 1). Further analysis by gender revealed significant correlations between the ZDS and PA in both males (R = −0.26, *p* = 0.037) and females (R = −0.52, *p* = 0.002). However, no statistically significant correlation was found between the ZAS and PA when analyzing the whole sample (R = −0.10, *p* = 0.31) (Figure 2), or separately by gender (Female: R = −0.23, *p* = 0.200; Male: R = 0.01, *p* = 0.960). A statistically significant correlation has been observed between the ZAS and ZDS (R = 0.57, *p* < 0.001).

**Figure 3:**
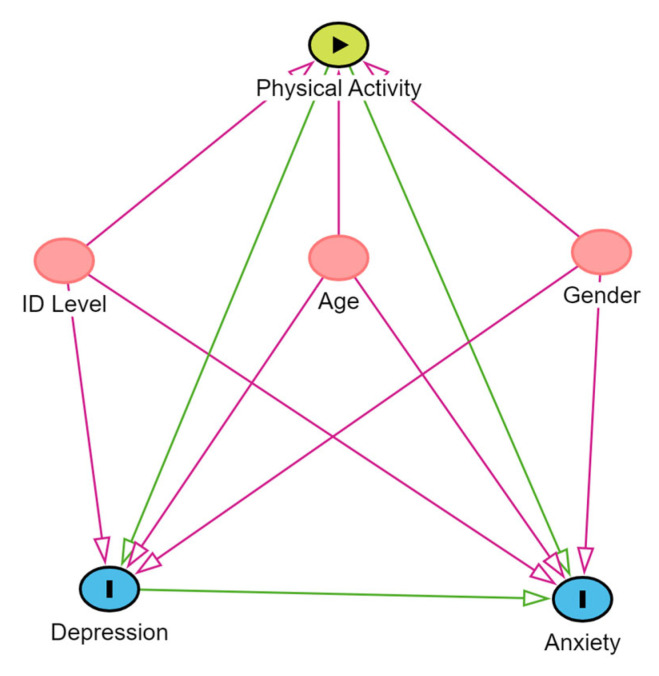
Directed acyclic graph (DAG) of the association between physical activity, anxiety, and depression. Legend: The variable in green is the exposure variable; those in blue were the outcome variables, and those in red are antecedents of the outcome and exposure variables. The figure shows only the variables that were selected for multivariate modeling based on the backdoor criterion.

Based on the directed acyclic graph (DAG) depicted in Figure 3, we applied a multiple linear regression model to predict the ZDS using PA, with exposure, age, gender, and intellectual disability level as covariates (minimal adjustment set). As reported in Table 2, the model significantly explains a substantial proportion of variance (R^2^ = 0.26, *p* < 0.001). Notably, the effects of PA (β = −0.10, *p* < 0.001), male gender (β = −3.56, *p* = 0.010), and intellectual disability level (β = −3.07, *p* = 0.008) were found to be negative and statistically significant. A similar analysis was conducted to assess the impact of PA on the ZAS. The model significantly explains a moderate proportion of variance (R^2^ = 0.18, *p* < 0.001). In this case, the effects of PA and age were not statistically significant (β = −0.03, *p* = 0.38 and β = 0.03, *p* = 0.58, respectively). On the other hand, male gender (β= −4.47, *p* = 0.003) and intellectual disability level (β = −3.23, *p* = 0.007) exhibited a negative and statistically significant impact.

### 3.3. Gender Differences in PA, Anxiety, and Depression

Participants demonstrated a generally active lifestyle, engaging in PA at an average of 1374 ± 1114 MET-min/week, with no discernible differences observed between males and females (*p* = 0.9, Wilcoxon rank sum test) (Table 1). Participation in team sports was significantly higher in males compared with females (*p* < 0.001, Fisher’s exact test). Significant differences were observed between males and females in anxiety levels (*p* = 0.040, Wilcoxon rank sum test), indicating higher reported anxiety levels among females compared to males. However, no statistically significant differences were found in self-reported levels of depression between the two groups. ANCOVA confirmed a significant main effect of sex on both outcomes, with men reporting lower scores than women (ZAS: β = −7.83 ± 2.61, *p* = 0.003; ZDS: β = −7.99 ± 2.52, *p* = 0.002). For anxiety, the association with PA did not reach significance (β = −0.09 ± 0.05, *p* = 0.067), whereas for depression, it was significant and negative (β = −0.18 ± 0.05, *p* < 0.001). Crucially, the PA × sex interaction was not significant for either the ZAS (β = 0.08 ± 0.06, *p* = 0.16) or ZDS (β = 0.11 ± 0.06, *p* = 0.058), indicating that the slope of the PA–mental health relationship did not differ between men and women. The full results are reported in the Supplementary Materials.

## 4. Discussion

The present study aimed to investigate the relationship between perceived mental health, PA levels, age, gender, and intellectual disability levels among adults diagnosed with intellectual disability.

### 4.1. Gender Disparities and Mental Health

Notably, our results revealed significant gender disparities in anxiety levels among participants, with females exhibiting higher levels of anxiety compared to males. However, no substantial gender-based differences were observed in the reported depression levels. These findings resonate with the existing literature [34], suggesting that females with intellectual disability might be more susceptible to experiencing anxiety-related symptoms than males within the same demographic group. The regression analysis highlighted significant and inverse associations between intellectual disability levels and both anxiety and depression scores. Previous studies suggested that people with mild intellectual disability are particularly vulnerable to anxiety and depression by virtue of their higher probability of long-term exposure to numerous social and environmental stresses and abuse [35,36].

These stressors may include increased dependence on caregivers, reduced autonomy in decision-making, limited access to inclusive social or vocational environments, greater exposure to stigma and exclusion, and lack of tailored psychological support [37]

Similarly, the male gender exhibited a negative association with the ZAS and ZDS, suggesting that gender plays a pivotal role in shaping mental health outcomes among people with intellectual disability. Gender does not exhibit a statistically significant difference in the amount of PA carried out, while males are more involved in team sports than females. However, adding team sport participation to the regression analysis does not improve the fit of the model, suggesting that being engaged in PA with high social involvement is not directly correlated with anxiety and depression symptoms. However, we cannot exclude that participants involved in individual sports could benefit from high social involvement during training, excluding the possibility of observing this phenomenon in the whole sample.

### 4.2. PA and Mental Health Associations

The negative association between physical activity and depressive symptoms observed in our study may be explained by several mechanisms. From a biological perspective, regular physical activity has been shown to modulate neurochemical systems involved in mood regulation, including increased availability of serotonin, dopamine, and endorphins [38]). Physical activity can also promote positive social interactions and reduce feelings of loneliness, especially in group or community-based settings. Additionally, engaging in regular activity may foster improvements in self-esteem, perceived competence, and self-efficacy, which are recognized protective factors against depressive symptoms [39]. These mechanisms may be particularly relevant for individuals with intellectual disabilities, who often experience restricted autonomy and limited opportunities for meaningful social engagement.

Contrary to expectations, the study did not find a significant direct association between reported anxiety levels and PA. This result is in contrast with previous research conducted on an analogous population [21], in which time spent in moderate-intensity PA was negatively associated with the occurrence of anxiety disorders. However, anxiety disorders were dichotomized as absent or present based on previous diagnosis without considering potential different levels of the severity of the disease. Nevertheless, a relationship emerged between depression and PA, indicating a negative and significant correlation. These results are in good concordance with the epidemiological study carried out on a younger population in a sports context, reporting that participation in high-level sports competition was associated with a 49% reduction in the risk of depression [40]. Our results showed a strong correlation between the ZAS and ZDS, suggesting that a co-occurrence of anxiety and depression symptoms can be observed analogously to what has been reported in the existing literature, in both general and clinical populations [41,42].

From a theoretical perspective, this study adds to the growing body of evidence identifying physical activity as a key determinant of mental health in individuals with intellectual disabilities, alongside demographic and clinical factors. From a practical standpoint, the findings support the development of targeted interventions to promote mental well-being in adults with intellectual disabilities, emphasizing the importance of considering relevant factors such as level of intellectual disability, age, and gender.

### 4.3. Limitations and Future Directions

Several limitations should be considered when interpreting these findings. First, the cross-sectional design limits the establishment of causality between variables. Second, the study did not encompass factors such as environmental influences or coping mechanisms that might contribute to mental health perception among individuals with intellectual disability. Third, on average, participants reported engaging in a high level of weekly PA, slightly surpassing the average levels found in other studies involving the same population [43,44,45]. However, it is important to consider that a portion of the participants were recruited from sports groups, potentially influencing these results. This aspect of the recruitment strategy limits the generalizability of our findings to people living in a clinical context or with a radically different level of social involvement. Fourth, the literature highlighted that adults with Down syndrome tend to be more physically active in comparison to individuals of similar age with other forms of intellectual disability [45]. Specifically, our sample comprises 74% of individuals with Down syndrome. Finally, the PA assessment relied on self-reported measures, which might have partially impacted the outcomes. Nevertheless, the measurement demonstrated good reliability, validity, and consistency with previous studies. Additionally, the data collection process was designed to minimize potential misunderstandings regarding the measurement instruments and was overseen by two of the authors to ensure accuracy. To enhance accuracy, future research should consider integrating objective tools, such as accelerometers or wearable activity trackers, to validate or complement self-reported data.

## 5. Conclusions

Despite these limitations, our study underscores the negative relationship between perceived mental health and PA in people with intellectual disability. These findings advocate for tailored interventions focusing on promoting physical exercise to improve mental well-being among individuals with intellectual disability. This study highlights gender differences in anxiety perception, confirming that females are more likely to experience anxiety compared to males. It emphasizes the importance of gender as a factor influencing these differences; nevertheless, gender does not appear to affect the relationship between physical activity and mental health in adults with intellectual disability.

Overall, this study provides valuable insights into the complex interplay between gender, intellectual disability level, PA, and mental health perception among individuals with intellectual disabilities. Future longitudinal studies exploring a broader range of influencing factors and intervention strategies could further enhance our understanding of mental health and overall well-being in this vulnerable population.

## Figures and Tables

**Table 1:** Participants’ characteristics and comparison between males and females.

		Gender		
Variables	Overall, N = 99	Female, N = 34	Male, N = 65	Adjusted *p*-Value
**Age**	33 (12)	37 (13)	31 (12)	0.079
**Height (cm)**	161 (12)	152 (7)	165 (12)	<0.001
**Weight (kg)**	64 (14)	57 (12)	68 (13)	0.004
**BMI (kg/m^2^)**	24.7 (3.8)	24.7 (4.8)	24.7 (3.3)	0.99
**Type of disability**				
Free Trisomy 21	58/78 (74%)	14/23 (61%)	44/55 (80%)	
Mosaic Trisomy 21	9/78 (12%)	4/23 (17%)	5/55 (9.1%)	
Oligophrenia	6/78 (7.7%)	3/23 (13%)	3/55 (5.5%)	
Other ID	5/78 (6.4%)	2/23 (8.7%)	3/55 (5.5%)	
Unknown	21	11	10	
**Intellectual Disability**				
Mild	29/89 (33%)	12/32 (38%)	17/57 (30%)	
Moderate	53/89 (60%)	17/32 (53%)	36/57 (63%)	
Severe	7/89 (7.9%)	3/32 (9.4%)	4/57 (7.0%)	
Unknown	10	2	8	
**Zung Anxiety Score**	35 (7)	38 (9)	33 (6)	0.041
**Zung Depression Score**	36 (8)	39 (9)	35 (6)	0.12
**Physical Activity (MET/min-week)**	1374 (1114)	1407 (1173)	1357 (1090)	0.97
**Participation in Team Sports**	47/99 (44%)	6/33 (35%)	41/65 (63%)	0.003
**Participation in Individual Sports**	50/99 (50%)	16/33 (48%)	34/65 (52%)	0.068

Data are presented as mean (SD) or frequency (%). BMI, body mass index; ID, intellectual disability; MET, metabolic equivalent.

**Table 2:** The results of the linear regression model. All continuous predictors are mean-centered and scaled by 1 standard deviation. The outcome variable is in its original units.

	Zung Depression Score			Zung Anxiety Score		
Predictors	Estimates	CI	*p*	Estimates	CI	*p*
Intercept	45.61	38.47–52.75	**<0.001**	43.05	35.68–50.42	**<0.001**
Physical activity	−0.10	−0.16–−0.04	**0.001**	−0.03	−0.09–0.03	0.387
Age	0.06	−0.05–0.18	0.291	0.03	−0.09–0.15	0.580
Gender male	−3.57	−6.41–−0.72	**0.015**	−4.48	−7.41–−1.54	**0.003**
ID level	−3.08	−5.33–−0.82	**0.008**	−3.23	−5.56–−0.89	**0.007**
R^2^/R^2^ adjusted	0.261/0.230			0.182/0.147		

## Data Availability

The datasets used and/or analyzed during the current study can be made available by the corresponding author on reasonable request..

## Supplementary Materials

The following supporting information can be downloaded at: https://www.mdpi.com/article/10.3390/jfmk10030285/s1.
