# Supplementary material for: How Does Physical Activity Affect the Mental Health of Adults with Intellectual Disability? A Cross-Sectional Study Analyzing the Complex Interplay Between Variables
Source: J Funct Morphol Kinesiol. 2025 Jul 24;10(3):285. doi: 10.3390/jfmk10030285 (PMC12371989; doi:10.3390/jfmk10030285)
Supplement: Supplementary file 1 [file jfmk-10-00285-s001.zip › jfmk-3700668-supplementary.pdf]

Results of D’Agostino–Pearson tests for normality across all study variables.

Variable	z-value	p-value
Age	1.934	0.053
Height	1.101	0.270
Weight	0.610	0.541
BMI	1.000	0.317
Type of Disability	5.167	<0.001
Intellectual Disability	0.461	0.644
Zung Anxiety Score	3.588	<0.001
Zung Depression Score	2.794	<0.001
Physical Activity	3.608	<0.001
Participation in Team Sports	-2.742	0.006
Participation in Individual Sports	-3.363	<0.001

ANCOVA model predicting Zung Anxiety Score (ZAS) from Physical Activity, Gender, and their interaction (PA × Gender).

Variable	Estimate	Standard Error	t value	P value
Intercept	40.96	2.12	19.24	<0.001
Physical Activity	-0.09	0.05	-1.84	0.067
Gender (Male)	-7.83	2.61	-2.99	0.003
Interaction Term	0.08	0.06	1.41	0.16

ANCOVA model predicting Zung Depression Score (ZDS) from Physical Activity, Gender, and their interaction (PA × Gender).

Variable	Estimate	Standard Error	t value	P value
Intercept	45.52	2.05	22.14	<0.001
Physical Activity	-0.18	0.05	-3.87	<0.001
Gender (Male)	-7.99	2.52	-3.16	0.002
Interaction Term	0.11	0.06	1.91	0.058

Overview of measurement instruments: item structure, scoring methods, and relationship to study constructs.

**Instrument**	**Construct**	**# Items**	**Example Item**	**Response Scale**	**Scoring Method**	**Score Range**	**Interpretation**
Zung SDS	Depression	20	"I feel down-hearted and blue"	1–4 Likert	Sum of items (some reversed)	20 –80	>50 = clinically significant depression
Zung SAS	Anxiety	20	"I feel more nervous and anxious than usual"	1–4 Likert	Sum of items (some reversed)	20 –80	>45 = clinically significant anxiety
IPAQ-SF	Physical activity	7	"How many days did you walk for at least 10 minutes at a time?"	Open numeric	MET-min/week (scoring protocol)	0 – infinite	Higher score = more activity

Note: Full scoring algorithms are available in the official manuals of the respective instruments.
